# Book Review

**Published:** 1915-11

**Authors:** 


### BOOK REVIEWS

EVE DORRE, The Story of Her Precarious Youth. By Emily Viele Strother. 256 pages. E. P. Dutton & Co., New York. $1.35 net.

This is a charming story of a young girl growing from childhood to motherhood in France though she was of American parentage and had much of the western point of view to begin with. It is not a trade novel written to order. It seems to be the spontaneous expression of the recollections of a girl's life, but such a girl! She is delicious all through and so unconventional that you know she is real and like the little sweetheart of long ago might have been if you had continued to know her and she had had a chance.

The story is fresh and free and good throughout and incidentally it shows the French life is clean and fine and that young girls are treated as pure and sweet despite the impression that salaciously minded travelers have tried to make to the contrary.

A postscript shows intense patriotism, but the story itself portrays a love of country that accounts for the patriotic expression under the present stressful circumstances, and it is the story we like.

---

EDUCATIONAL HYGIENE from the Pre-School Period to the University, edited by Louis W. Rapeer, Ph.D., Professor of Education, Pennsylvania State College. Illustrated. 645 pages. Charles Scribner's Sons, New York, Chicago and Boston.

The term Educational Hygiene is a new one and is intended to comprise all that has been done hygienically in regard to the child at school as well as the advice given to parents concerning home care, and now to this is added the teaching of advanced students what they should know in order to manage schools properly themselves. As the author says in his preface, "the value" is an attempt to bring together in organized form the latest information and advice of leading specialists in all the large phases of the subject." At the same time reference is made to many monographs that might extend the reader's information in detail.

The book has four parts: Health Sociology, The Administration of Educational Hygiene, The Divisions and Practice of Educational Hygiene and The Hygiene of the College.

The School Sanitation standards thus collated and established are invaluable and the extent of the field through which the reader is taken is astonishing. A wealth of suggestions is lavished upon the student and at the same time he is made to realize that no longer will hit or miss methods be tolerated in the handling of children and adolescents when the responsibility for their development is reckoned. The illustrations are excellent and numerous, and the construction of the book is good and convenient. It should be in the hands of everyone interested in social work and thoughtful of children's needs.

---

THE NOSE, THROAT AND EAR, Their Functions and Diseases. A Treatise upon The Breath-Road, Food-Road and Accessary Organs by Ben Clark Gile, M. D., Instructor in Otology in the University of Pennsylvania, Assistant in the Department for the Nose, Throat and Ear and Dispensary Chief at the Presbyterian Hospital; Consulting Laryngologist to the Taylor Hospital; Fellow of the American Larygological, Rhinological and Otological Society. 456 pages. 131 illustrations, 8 in colors. Cloth. Philadelphia, P. Blakiston's Son and Co.

It is the expressed object of the author to simplify and arrange the material literature and experience furnishes upon his subject so that one may read systematically and consecutively instead of doing hit and miss work and having to sort things out later one. He has accomplished this and at the same time, he has eliminated much that goes to make a volume large and imposing and burdensome. The book is convenient in size. Yet the book does not give the impression of being merely an epitome from which one must turn to more extended works for complete information. It tells what the author's ideas are as to the last thing in his subject and it tells it well. The matters in the title are exhaustively covered.

In the methods of the specialist there is great variety and so several of the things he advocates as to parphernalia and instruments would not suit us, but this is a matter of personal equasion and any one competent to read this book would already have had his own ideas partly formed at least. But simplification and directness make the dominant chord of the chapters and they harmonize the ideas all through the book. In tonsillectomy, he speaks of the "craft-master's" technique. An assistant is of help, of course, else he would not be an assistant, but tonsillectomy can be done nicely by two men, the operator and the anaesthetist without a nurs. We can not forbear to protest against the use of cocaine as advocated in diseases of the lingual tonsil.

It isn't necessary, and the drug is too dangerous ever to use unless the demand for it is surgically imperative.

The Chapter on Internal Ear Diseases contains the best summing up and the plainest exposition of our knowledge of this subject that we know of; and the following chapter on Intracranial Complications of Ear Diseases is equally attractive. The work that has recently been done in Philadelphia in these important studies is clarly presented and credit given.

The author and publishers are to be congratulated upon the book.

---

APPLETON'S MEDICAL DIRECTORY, Edited by Smith Ely Jeliffe, A. M., M. D., Ph.D., Adjunct Professor of Diseases of the Mind and Nervous System, New York Post Graduate Medical School, Assisted by Caroline Wormeley Latimer, M.D., A. M., formerly Instructor in Biology Women's College, Baltimore. New York and London, D. Appleton & Co. 1915. 8vo., 945 pages. Flexible leather cover.

It has been the object of the editors to select and arrange all the words in present professional use so as to eliminate those that are of long past value and to hold those we continue to use as nearly as possible to their proper application. While doing this they are fully aware of the constant growth and great flexibility of our language and the requirements of discoveries for adequate expression.

So far as one can test a dictionary within the brief time given for a review, it would seem that they have fulfilled their purpose and this gives one confidence to rely upon the book for future assistance and enlightenment.

There are no illustrations in the text and there is nothing to divert attention when seeking a word. It can be found readily and the definition is as brief and pointed as the word will permit. There are no diacritical marks, but the pronunciation is indicated by phonetic spelling directly after every word needing it.

The appendix contains sections on Analysis of Body Fluids, Dietary, Bottle Fed Babies, Common Poisons, Weights and Measures, Reciprocity, Suggestions to Medical Writers, etc.

The book is of convenient desk size and its flexible covers and clear typing on unglazed paper make it a desirable companion.

---

THE STORY OF CANADA BLACKIE, by Anne P. L. Field, with an introduction by Thomas Mott Osborne, Warden of Sing Sing Prison. New York, E. P. Dutton and Co. Cloth, 157 pages. $1, net.

This is a remarkable little volume that tells a wonderful story so fascinating that one can not leave it until it is finished, and then he wants it near him to refer to and to quote to his friends. It is up-to-date in a special sense because it treats of the problem of the care of the imprisoned criminal which all the world knows that Osborne is doing his best to solve. Mr. Osborne does not claim that there is anything new for his methods, but he does want thinking men and women to comprehend what he has done and is planning in the application of the humanitarian and economic principles to the salvation of criminals as this idea is placed over and against the awful failures which the purely punitive and revengeful methods have produced.

This document which the authoress has constructed with discriminating sympathy is the story of a bad man who made good. Not only that but he has told about it in a series of letters that make up the bulk of the book. He was an educated man in the beginning and has written nothing mauldin, tho we would suggest to readers that it is well to remember when studying his letters that his surroundings had deprived him of society and that the introspective life can not fail to show its mark upon every prisoner no matter what he does.

There is a ring of truth and conviction about the letters that does away with any sentimentality that might otherwise be confusing. To those of us who have been familiar with the perplexities of the malingerer both in his sickness and his repentance while in custody, these letters are "right." They could not be invented. One reading them can safely give way to the emotions they induce and be the better able to shape his conduct afterward.

To the physician, they should be of special interest because they will open the eyes of all men who have not handled convicts, to the subject that must soon engage all our communities, and they will make each doctor better prepared to lead public opinion along a line that has as much to do with his profession as the prevention of any great ill of humanity. Not only should the doctor of the county lead in this matter, but the family doctor who studied the kindly words of Mr. Osborne to Blackie, can use them with even greater effect in talking with a youngster who has some wrong ideas and needs advice before he has acquired the penitence to go to his father or the priest.

The book was probably sent to THE JOURNAL for review by mistake, for it is not medical in character, but we are enthusiastic over it for its interest and sociologic value and commend it accordingly.

---

THE BABY'S FIRST TWO YEARS, by Richard M. Smith, M. D., Assistant in Pediatrics Harvard Medical School, Assistant Visiting Physician, Children's Medical Department, Massachusetts General Hospital, Boston. Boston and New York, Houghton-Mifflin Co., The Riverside Press, Cambridge, 1915. Cloth. 156 pages. Several illustrations and charts. 75 cents, net.

This little book takes up in a charming manner the life of the baby during his first two years and tells how to meet and prevent most of the troubles which tradition would say he was heir to. And when the troubles have come in spite of or without preventive care, it shows the way out. There is nothing specially new claimed for it but it is in touch with the last thing for the good of the baby and tells about it so nicely that it would be of great value in the hands of a mother who has several "in-laws" and others experienced in wrong-doing and sentimentality around the house to bother her.

The book will refresh the doctor's memory and it migh save him a lot of trouble if he made a present of it to each of the ladies he delivers. It would not be a case of a little knowledge being a dangerous thing because the book is too well thought out for that.

---

THE TREATMENT OF FRACTURES: With Notes Upon a Few Common Dislocations. By Charles L. Scudder, M. D., Surgeon to the Massachusetts General Hospital; Associate in Surgery at the Harvard Medical School. Eighth Edition, Revised and Enlarged. Octavo volume of 734 pages, with 1057 original illustrations. Philadelphia and London: W. B. Saunders Company, 1915. Polished Buckram, $6.00 net; Half Morocco, $7.50 net.

This book covers all classes and types of fractures. Of nearly any conceivable fracture one may have the physician can find diagrams and discussions covering the same or similar conditions. Equally good for class work or for reference and well worth any man's time to carefully review it. D. Y. S.

---

A PRACTICAL TREATISE ON DISEASES OF THE SKIN. By Oliver S. Ormsby, M. D., Professor and head of the Skin Department of Skin and Veneral Diseases, Rush Medical College, Etc. 303 engravings and 39 plates in colors and monochrone. Published by Lea & Febiger, Philadelphia.

Ormsby has presented in this volume a sufficiently concise yet comprehensive survey of skin diseases with the important advances made during the past few years. The newer methods of diagnosis and treatment of proved value have been included with the results of research in etiology and pathology. Dermatological literature has also been carefully revised in the preparation of this excellent treatise. The illustrations are exceptionally good.

---

THE PHYSICIAN'S VISITING LIST (Lindsay & Blakiston's) for 1916. Philadelphia, P. Blakiston's Son & Co.

This List has been published for sixty-five years and is familiar to most men in the profession. It is arranged for 25, 50, 75 and 100 patients per day or week in different sized books and in the higher numbers, there are two volumes so that the pocket size can be maintained. There is also an arrangement for perpetual use in another volume.

The books are handsomely bound in leather and have a tuck, pocket and pencil and all are furnished with memorandum spaces, as well as the regular recording pages, calendars, etc.

The prices vary from $1.25 to $2.50, according to the size.

Booksellers and Druggists have them on sale.

